# P-1601. Reducing Inappropriate *Cytomegalovirus* and Hepatitis Genotype Testing with Diagnostic Stewardship

**DOI:** 10.1093/ofid/ofae631.1768

**Published:** 2025-01-29

**Authors:** Christian Darren Baguistan, Regina Wulff, Stacia Semple, Sonal Kumar, Lars Westblade, Harjot K Singh

**Affiliations:** Weill Cornell Medical Center, vallejo, California; New York Presbyterian Hospital, New York, New York; Weill Cornell Medical Center, vallejo, California; Weill Cornell Medical Center, vallejo, California; Weill Cornell Medicine, New York, NY; Weill Cornell Medicine, New York, NY

## Abstract

**Background:**

Over testing is a common problem that impacts patient safety and quality. Less frequent, yet expensive tests, such as CMV, HBV, and HCV resistance testing are frequently ordered inappropriately. Inappropriate ordering of these tests can lead to waste (unused test), false positives, unnecessary cost. Diagnostic stewardship promotes the judicious use of tests to enhance patient care and curb unnecessary expenses.

**Table 1:**
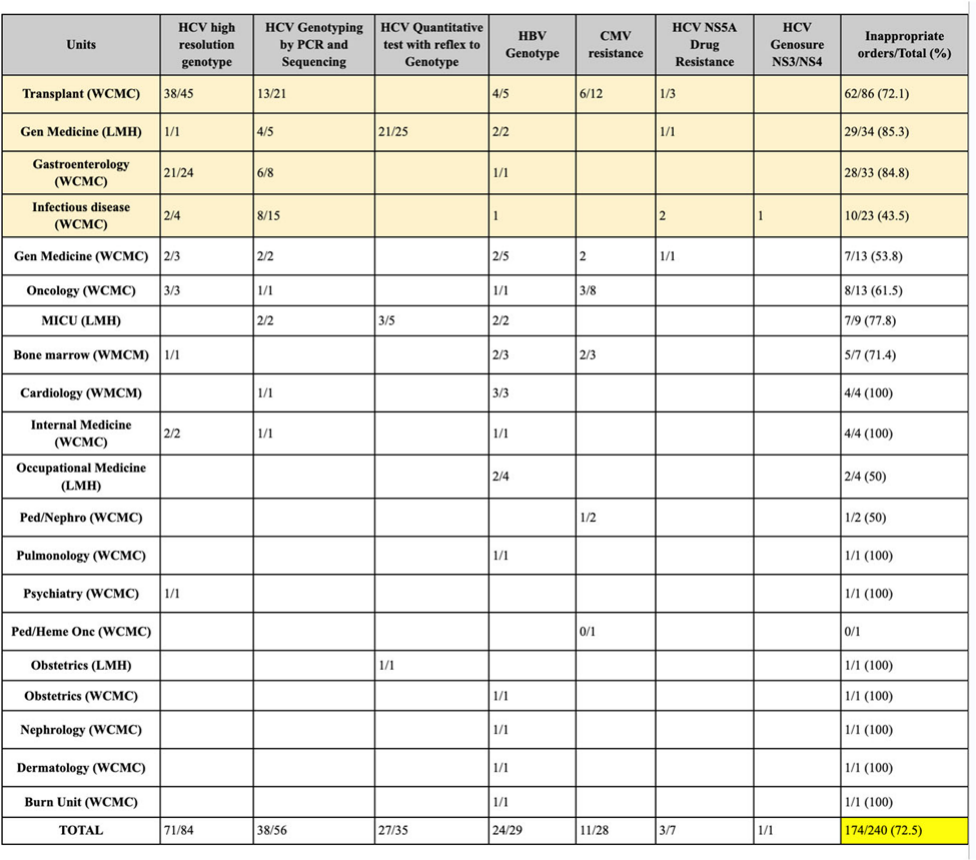
Summary Inappropriate/Total # of tests per Medical unit and Campus

Number of inappropriate vs total number of orders per unit and campus during 2022 (retrospective data)

**Methods:**

We performed a Quality improvement study (IRB exempt) from 12/2023 – 4/2024 using iterative PDSA cycles and compared data to 2022 baseline data. An interdisciplinary team conducted prospective review of all CMV, HBV, and HCV resistance tests at 2 NYC hospitals to determine appropriateness using established IDSA and AGA guidelines. All specimens were stored for up to 72 hours during review which included outreach to ordering providers when chart indication was not clear. Only providers cancelled tests if they agreed on inappropriateness. Baseline data were assessed for appropriateness using only patient charts.

**Table 2:**
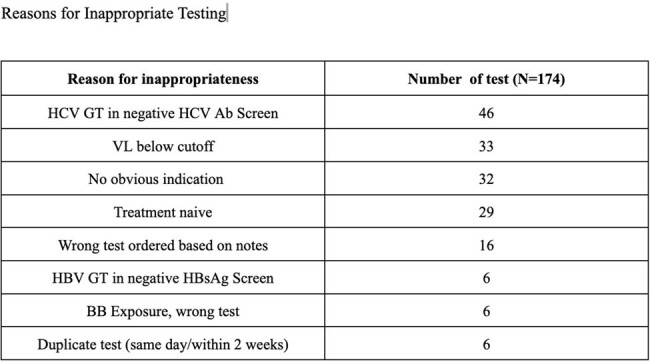
Reasons for Inappropriate Testing Summary of Reasons for Inappropriate Testing during retrospective review

**Results:**

We reviewed 240 tests in the baseline period and 33 tests to date in the intervention period. Of the baseline period, 73% met inappropriateness definition. (Table 1) Reasons for inappropriate testing are shown in Table 2. With prospective review, 18/33 tests met definition of inappropriate, of which 12/18 were cancelled by providers, (Figure 1) with reduction in inappropriate testing to 28% (Figure 2).

**Figure 1:**
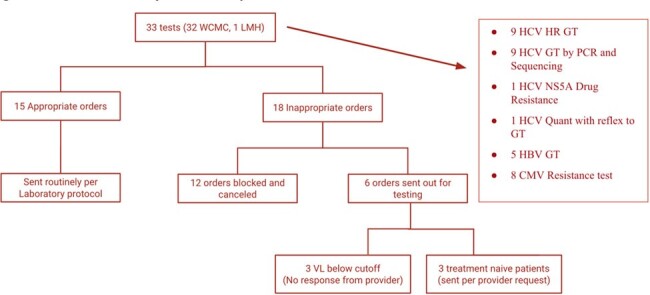
Summary of Prospective Audit and Feedback orders Summary of the total number of appropriate vs inappropriate genotype orders during prospective review

**Conclusion:**

In this QI project with prospective audit and feedback, we were successful in reducing inappropriate resistance testing. Based on the pattern of inappropriate testing, multiple opportunities for sustained interventions were uncovered including clinical decision support to reduce duplicate testing and need for reflex testing.

**Figure 2:**
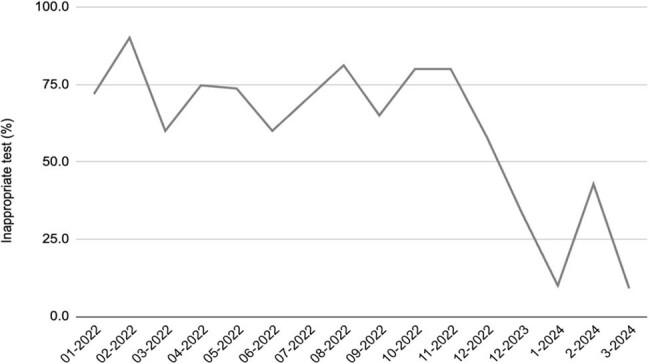
Run chart of Rate of Inappropriate test orders (Retrrospective and Prospective data) Rate of inappropriate test orders during retrospective review (72.5%) vs prospective review (28.6%)

**Disclosures:**

**Sonal Kumar, MD**, Cymabay: Advisor/Consultant|Gilead Sciences: Honoraria|Ipsen: Advisor/Consultant|Madrigal Pharmaceuticals: Grant/Research Support|Madrigal Pharmaceuticals: Honoraria|Novo Nordisk: Advisor/Consultant|Novo Nordisk: Honoraria **Lars Westblade, PhD**, Accelerate Diagnostics, Inc: Grant/Research Support|bioMerieux, Inc: Grant/Research Support|Element Materials Technology: Grant/Research Support|Hardy Diagnostics: Grant/Research Support|Roche Molecular Systems, Inc.: Advisor/Consultant|Roche Molecular Systems, Inc.: Grant/Research Support|Selux Diagnostics, Inc.: Grant/Research Support|Shionogi, Inc: Advisor/Consultant|Talis Biomedical: Advisor/Consultant

